# Cancer patients’ experiences with immune checkpoint modulators: A qualitative study

**DOI:** 10.1002/cam4.2940

**Published:** 2020-03-02

**Authors:** Kari Ala‐Leppilampi, Natalie A. Baker, Chris McKillop, Marcus O. Butler, Lillian L. Siu, Anna Spreafico, Albiruni R. Abdul Razak, Anthony M. Joshua, David Hogg, Philippe L. Bedard, Natasha Leighl, Amit M. Oza, Janet A. Parsons, Aaron R. Hansen

**Affiliations:** ^1^ Applied Health Research Centre Li Ka Shing Knowledge Institute St Michael's Hospital Toronto Canada; ^2^ Turalt, Inc Toronto Canada; ^3^ Princess Margaret Cancer Centre University Health Network Toronto Canada; ^4^ Division of Medical Oncology and Hematology Department of Medicine University of Toronto Toronto Canada; ^5^ Department of Medical Oncology Kinghorn Cancer Centre St Vincents Hospital and Garvan Institute of Medical Research Sydney Australia; ^6^ Department of Physical Therapy and the Rehabilitation Sciences Institute University of Toronto Toronto Canada

**Keywords:** costimulatory antibodies, Health‐related quality of life, immune checkpoint inhibitors, immunotherapy, patient experiences

## Abstract

**Background:**

Minimal qualitative data exist on the experiences of cancer patients treated with immune checkpoint inhibitors or costimulatory antibodies. Understanding the day to day experiences of patients being treated with immune checkpoint modulators, and how these relate to their health‐related quality of life, can inform future research and lead to better clinical decision‐making and care. We report here the first in depth qualitative study to consider patients' diverse and complex experiences with immune checkpoint modulators, with a focus on side effects and how these impact daily life.

**Methods:**

This single‐center qualitative study was based on focus groups and semistructured interviews. Patients who were being treated or who had been treated with immune checkpoint modulators within the last year for a range of cancer diagnoses were recruited. Interpretive description informed our inductive, iterative approach to analysis.

**Results:**

Eight themes were identified, characterizing the complexity of these patients' lived experiences: major categories of side effects experienced and how they impacted patient well‐being; the heterogeneous nature of side effects experienced; living with uncertainty; reframing the meaning and severity of SEs; focus on survival, hope, and being positive; acceptance and adaptation; feeling supported; and faith in medical innovation. Throughout their accounts, participants highlighted the profound impact that immune checkpoint modulators had on their daily lives.

**Conclusion:**

This is the first in‐depth qualitative study into patient accounts of their experiences of treatment with immune checkpoint modulators, related side effects, and how it impacted their daily lives. This research is an integral initial step in developing an instrument that will assess treatment‐related side effects in patients treated with this form of therapy.

## Graphical abstract

This is the first report of patients' accounts of their experiences with ICM treatment, related SEs, and its impact on daily life. This research is an integral initial step in the development of an instrument that will measure treatment‐related SEs in patients treated with ICMs.

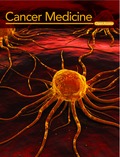

## 1 BACKGROUND

Treatments modulating immune checkpoints such as costimulatory agonists and checkpoint inhibitors can produce antitumor immune responses with the potential to improve clinical outcomes for cancer patients. Several checkpoint inhibitors have received regulatory approval and are now standard of care for certain solid tumors.^1^, ^2^ While extensive qualitative research exists on the experience of patients receiving anticancer therapies, such as chemotherapy or endocrine agents,^3^, ^4^ there is limited qualitative research on patients' experiences with immune checkpoint modulators (ICMs).

ICMs have different mechanisms of action when compared to standard anticancer therapies such as chemotherapy and radiation. Multiple studies have reported a unique constellation of side effects (SEs), referred to as immune‐related adverse events (irAEs).^5^ These SEs are mediated through the immune system and can affect any organ or tissue over the course of, or even after the completion of treatment; they range from mild to life threatening and can occur at any time during or after therapy.^6^, ^7^ Common and important irAEs include rash, itch, fatigue, dyspnea, cough, diarrhea, abdominal cramps, and rectal bleeding to name several key symptoms.^8^ ICMs may have also nonimmune‐related SEs and sometimes these can be difficult to differentiate from irAEs. Typically irAEs are reported to be mild, well tolerated and easily managed for most patients, although a small fraction may experience severe irAEs that in some cases are fatal. Complicating this picture, ICMs may be administered to patients over a long period raising the possibility that even mild irAEs may have a cumulative impact on patients and their health‐related quality of life (HRQOL). Finally, the treatment of irAEs often involves prolonged courses of corticosteroids and or other immunosuppressants,^8^, ^9^ which can have their own set of SEs and there are some distinct toxicity management considerations compared with other anticancer therapies.

We conducted this qualitative study as part of a broader series of investigations to develop a treatment toxicity subscale for ICM SEs. The specifics of the subscale's development have been described in a separate publication^10^ and detail the processes we undertook in terms of item generation, reduction, and scale refinement. Results of a systematic review^7^; as well as information from physician surveys and interviews, patient interviews, and cognitive debriefing were all used to identify a list of potential items for inclusion into this scale.^10^ The qualitative investigation outlined here provides an in depth exploration of patients' first‐hand experiences with ICMs, their SEs, and how these impact their daily lives.

## 2 METHODS

This study employed qualitative methods as they were best suited to provide a comprehensive understanding of how cancer patients experienced ICM treatment, and how they felt it impacted their daily lives. The overarching approach was interpretivist^11^ and our methodology was that of interpretive description.^12^, ^13^ The study (NCT02651831) was reviewed and approved by the University Health Network Research Ethics Board. Patient interviews and focus groups were conducted between August 2015 and March 2017.

### 2.1 Sampling

Patients were recruited from Princess Margaret Cancer Centre, a large academic hospital specializing in cancer care, and located in Toronto, Canada. Eligible patients were proficient in English, 18 years and over with advanced, incurable cancer who had been treated with ICMs as standard of care or as part of a clinical trial. Potential participants were first approached by their treating medical oncologist or by the principal investigator (AH) at the request of the treating team, to see if they were interested in participating. Those who expressed an interest in participating were then contacted by a member of the research team who explained the study in greater detail. Written consent was obtained for all of those participating in focus groups and in person interviews, while verbal consent was obtained from those participating in telephone interviews.

We employed a purposive sampling approach wherein efforts were made to recruit ICM patients with varying demographic, diagnostic, and treatment characteristics in order to elicit a wider range of patient experiences.^14^ For example, participants with different cancer types; of different ages; those with known SEs that were rare or severe; those treated with different classes of ICMs; and participants at different phases of their ICM treatment (thereby capturing early, late, and posttreatment effects) were recruited. In keeping with qualitative methodology, sampling was analytically driven, in that data collection was deemed complete when additional interviews failed to yield additional insights (thematic saturation).^15^, ^16^ Sample size was also guided by conventions for focus groups (ideally five to eight participants per group) and for interviews with heterogeneous groups.^11^, ^17^

### 2.2 Data collection

Both focus groups and in depth semistructured interviews were conducted with unique participants. Focus groups were conducted in person and interviews were conducted primarily by telephone unless participants requested in person interviews, which was permitted as per protocol. In person interviews took place in a private room adjacent to the clinic. All participants were provided with a $25 gift card, as a token of appreciation, and those in the focus groups were provided with complimentary refreshments. No refreshments were provided during in person interviews. Focus groups and interviews were conducted by team members with expertise in qualitative research methods (KA, NAB, and CM).

Focus groups were conducted first in order to quickly and efficiently provide the researchers with a cross section of numerous patient experiences, which then informed specific topics to be explored in greater depth within subsequent individual interviews. While focus group discussions can stimulate new ideas and reveal social dynamics, they may also deter some patients from disclosing certain personal experiences, details, and views as a result of the limited privacy and time available to each participant.^18^ Individual interviews offer participants greater privacy for discussing sensitive topics, and more time to share their experiences and views in greater depth.^19^

Combining focus groups and interviews afforded the opportunity to benefit from the strengths of both methods. This combined approach allowed us to elicit a wider range of perspectives, and to establish greater confidence in our ultimate findings through a triangulation of methods.^16^ Combining telephone and in‐person data collection strategies is acceptable practice in qualitative research, so long as issues of interview quality are accounted for by the interviewers and analysts.^20^, ^21^ Organizing focus groups was difficult because of scheduling issues and the health concerns of some patients. The telephone option was more flexible permitting more patients to participate in the study.

Semistructured interview guides were used for both the focus groups and individual in‐depth interviews. The guide began with an open‐ended question intended to allow the participants to describe their experiences in their own words, “Please tell me about your experiences with cancer, its treatment, and your most recent experiences with ICM treatment in particular.” While patients were encouraged to discuss their experiences with ICM agents, the guide ensured that certain topics were addressed. Patients were asked to describe their general experiences with cancer and prior cancer‐related treatments, and to discuss how the latter compared with their ICM treatment. They were also asked to describe all ICM‐related SEs they experienced and probed as to their impact on four HRQOL domains (physical, emotional, functional, and social). For example, four separate queries asked “How have the SEs you've described impacted upon your: strength, comfort or pain level; mood and state of mind; mobility and general ability to do daily tasks; relationships with family and friends?”

Both focus groups and interviews were audio recorded and transcribed verbatim, and then imported into QSR International's NVivo software (version 11), which was used to assist in qualitative data management and analysis.^22^

### 2.3 Data analysis

An analytic approach of interpretive description was adopted.^23^ This approach was inductive and iterative, to understand the experiences and impact of SEs from the patients' perspectives. The focus was the meanings patients attributed to situations and events, and the specific language they used to describe their experiences.^24^ The four domains of HRQOL served as general “sensitizing concepts” that provided a sense of reference and guidance as we interrogated the data, but they did not restrict our inquiry.^16^

Initially the transcripts were read multiple times and text was coded descriptively.^25^ Codes were then grouped and categorized according to the key themes identified as they related to patients' experiences with ICM treatment, the SEs and their impact on their daily lives. An iterative process of constant comparison was applied wherein themes were generated and continually tested and revised based on new readings and interpretations of the original transcripts.^26^

Techniques for ensuring analytic rigor included the use of multiple data sources (focus groups and interviews), multiple analysts, and ongoing research team meetings where the analytic framework and emergent themes were continually reviewed. The research team met formally every 3‐4 months with additional meetings taking place between the primary analyst (KA) and other team members (JAP, NAB). The research team included clinical members with extensive experience in caring for patients treated with ICM agents and qualitative research experts in studying cancer patients' treatment experiences and HRQOL. Consultation with an outside expert on cancer patients' experiences helped to provide additional perspective on our analysis.

## 3 RESULTS

Thirty‐seven patients participated and were enrolled from August 2015 to March 2017:14 took part in one of three focus groups (n = 6, 6, and 2), and 23 were interviewed individually (21 by telephone and 2 in person). Participant characteristics are described in Table 1. Focus groups lasted 52 to 80 minutes, and individual interviews were between 34 and 115 minutes in duration. Eight themes were identified from our analysis: major SE categories and impacts on HRQOL; heterogeneous experiences with SEs; living with uncertainty; reframing the meaning and severity of SEs; focus on survival, hope, and being positive; acceptance and adaptation; feeling supported; and faith in medical innovation.

**Table 1:** Patient and treatment characteristics

Characteristics	N = 37
Gender (Female:Male)	13:24
Age (years)	
Median	60
Range	24‐85
Tumor classification	
Endocrine	2
Gastrointestinal	3
Genitourinary	3
Gynecology	4
Head and Neck	2
Lung	1
Melanoma	18
Sarcoma	4
ICM Type (checkpoint target)	
PD‐1/PD‐L1 (inhibitory)	34
CTLA4 (inhibitory)	16
CD40 (stimulatory)	1
GITR (stimulatory)	1
ICOS (stimulatory)	1
OX40 (stimulatory)	1
ICM as single agent	22
ICM in combinations	17
Agents given in parallel:	
Immune therapy	8
Nonimmune therapy	9
Number of lines of ICM treatment	
1	25
2	11
3	1

### 3.1 Major SE categories and impacts on HRQOL

While participants described a wide range of diverse SEs, seven major categories were identified. Patients reported that these SEs affected their physical as well as emotional well‐being. In addition, they described the major impact SEs had on aspects of daily functioning such as employment and relationships with family and friends. ***Fatigue*** was the most frequently cited SE, but experiences varied greatly between participants. Roughly half reported feeling a bit tired for 1‐3 days after their infusion and did not report any significant impact upon their HRQOL. The other half reported severe and longstanding fatigue that was unlike anything they had experienced before and limited their ability to engage in recreational pursuits, household tasks, and personal care. As one participant described, “It was just a deep tired feeling that I am not even sure what word could describe it.” (Interview Participant [IP]7) Fatigue was the most commonly cited barrier to pursuing employment and was described as transforming family roles and dynamics. Some reported feeling guilty, discouraged, frustrated, angry, and worthless as a result of a lack of energy and motivation, and described how this affected their sense of identity as active and productive people. As one reflected, “I was once such a go getter.” (IP3).

***Gastrointestinal*** issues were the second most discussed category of SEs. Diarrhea was the most common symptom, ranging from mild to severe and often accompanied by stomach cramps. Participants described avoiding social situations, or having to take special precautions (eg, using incontinence products and staying near washrooms) due to worry about their diarrhea or frequent bowel movements. Severe cases of diarrhea resulted in steroid treatment and/or suspension of ICM therapy. Admissions to hospital for colitis‐like symptoms were described as being particularly lengthy (ie, 10 + days), arduous, and disruptive to participants' lives.

***Cutaneous*** SEs were also identified and were commonly associated with pain or discomfort. Itchiness, especially in the legs, was experienced with annoyance and frustration. As one participant described, “My calves would itch to the point that you could almost draw blood. They were just extremely, extremely itchy.” (IP8) Visible rashes or edema were reported and caused some participants to feel self‐conscious or to avoid social situations; as one participant explained, she did not want her friends to see her “that way”. (IP12) A few participants described experiencing very sudden, painful, and alarming whole body rashes that required hospitalization; these dramatic episodes caused participants to worry about the long‐term impacts of ICMs. Other participants described experiencing skin that was dry, callused, or sensitive to temperature, sunlight, or touch, which sometimes limited their activities (eg, prevented them from going outdoors) and/or required that they constantly apply lotions. Finally some participants reported experiencing spontaneous and painful burning sensations in the skin of the arms or feet.

***Musculoskeletal*** SEs were reported, including joint inflammation, swelling, pain, and stiffness affecting their upper and lower extremities. Muscular pain, soreness, weakness, and cramping were also reported. Some spoke of it as a more general body pain, “I have terrible pains in my legs, my feet ‐ I could hardly step on the pavement. I felt that my body was aching. I was away for a weekend and I couldn't get up these stairs and I am thinking my goodness…when I go to bed at night I am in bed and I ache, my legs, my feet, my back, everything aches.” (IP9) As a result of musculoskeletal SEs participants took more breaks when walking or doing household tasks, avoided sitting for long periods, used mobility aids, and adopted more sedentary lifestyles. Some commented that they felt that this aged them prematurely.

A number of participants recalled issues with ***nutrition and metabolism***, namely, loss of appetite and in some cases dramatic weight loss. Most described a change in their sense of taste, with food seeming too sweet, salty, tasteless, or simply “not right” (IP7). They reported missing the simple enjoyment and comfort they once derived from eating and the opportunities it provided to socialize with family and friends.

A number of participants described issues with ***abnormal body temperature***. They described repeated bouts of fever, or an ongoing low level “running fever” (IP8) that was punctuated by periodic spikes. In some cases fevers were described as being one component of a syndrome of flu‐like symptoms, which included chills, weakness, dizziness, headaches, and muscle soreness. Some reported being hospitalized for fever, receiving treatment in the form of fluids, steroids, or antibiotics. In some cases they experienced sudden and intense bouts of fever immediately following treatment, which could last many hours, or even days “Within half an hour of leaving hospital I was quaking with a fever of 104.5 and went from shuddering shakes to chills, to burning hot and it was all I could do to not pass out. We came home and I went into 4 straight days in bed with a raging 104.5 degree fever.” (Focus Group [FG] 2 Participant [P] 8).

Finally, participants also reported ***respiratory SEs*** *.* These included respiratory infections and congestion, which made breathing and/or vocalizing more difficult; one participant described it as akin to “a long and lingering cold.” (IP7). Some described dyspnea which forced them to pace themselves and to take more breaks during daily activities. In two cases participants described being treated for pneumonitis and pleural effusion.

### 3.2 Heterogeneous experiences with SEs

Despite commonality in terms of the general categories of SEs identified by participants, an overarching theme was **heterogeneity** in terms of their actual experiences. Participants reported a diverse range of side effects which did not all fit in the major categories identified, and the constellation of side effects experienced by any one participant varied widely. As is evident even within our description of SE categories above, patients often described the very same SEs with considerable variability with respect to onset (during or after treatment), timing (acute or delayed), duration, pattern (sudden, gradual, continuous, intermittent), severity, and impact upon their daily life. Some patients reported no to very few SEs, with minimal to no impact on their quality of life. Others described multiple and/or life threatening SEs. As one focus group member who suffered severe SEs commented on the diametrically opposite experience of another, “We are like bookends.” (FG1P4).

### 3.3 Uncertainty

Concerns about the diversity and unpredictability of SEs, permanence of SEs, long‐term health implications, and cessation of ICM treatment due to SEs were an ongoing source of anxiety for some patients. One described “always waiting for the next shoe to drop” (FG2P10), in terms of what the next SE would be. Another reported being unable to enjoy daily life as a result of a hypervigilance about her body, which was reinforced by the unpredictability of SEs and their treatment team's instructions to report any and all SEs. “If you are spending an inordinate amount of time dealing with physical issues and your mind is preoccupied with all of that sort of stuff, that really impacts on your quality of life. What ends up happening is that you become so internally focused that you don't really care or have time to look at what's happening out in the world and that's the question. What kind of quality of life is it then? I don't think that's really healthy to be sort of focused on what's going on inside all the time but I have never been more aware of bodily functions and how I feel physically.” (IP12) One participant characterized this living with uncertainty as follows: “…what kind of SEs am I going to get down the road? Because all of this stuff is so new, so they don't really know right? What's going to happen to me in another 15 years from all these drugs?” (IP1) Those who had treatment suspended because of SEs described anxiety about limited future treatment options and, in some cases, concerns over being disconnected from their ICM treatment team. One participant described his suspension of treatment as a “very low point” as he felt “orphaned” and was left “floating”. (IP20).

### 3.4 Reframing the meaning and severity of SEs

Participants sometimes reframed their SEs in their accounts to minimize them or question their link to ICMs. They said they considered ICM SEs as being far less severe than toxicities from other treatments such as chemotherapy and radiation and attributed SEs to other causes such as prior treatment, preexisting medical conditions, tumor progression, or aging. As one participant described, “It's really hard to tell like what your other treatment is doing, what your aging body is doing, what cancer is doing and what just the [ICM] med is doing.” (IP6) A number of participants suggested that certain SEs (eg, muscle weakness, swelling, stomach pain, fatigue) were related to the steroids used to manage ICM‐related SEs. Some participants interpreted SEs as a positive sign that the ICMs were working, suggesting that this was indicative of an activated immune system. This interpretation was portrayed as a source of comfort for participants. As one participant described*,* “And the other thing we found as we were going through the sessions was if you get some side effects it's a good sign that the drug is actually working.” (IP11).

### 3.5 Focus on survival, hope, and being positive

Most participants were focused on survival and glad to have received ICMs, regardless of SEs or treatment failure. As many felt they were out of options prior to ICM treatment and essentially had “nothing to lose”, it was described as providing an important source of “hope” (FG2P10). Those who experienced treatment success viewed this as offsetting any negative impact from SEs, “I got every SE in the book…but the main thing is that it worked.” (IP4) Participants emphasized the importance of maintaining a positive attitude because they felt this influenced treatment outcomes, and extended this to their experience of SEs. As one explained, he never spoke of “pain” in reference to his joints as that would be “too negative”; instead he used the term “discomfort”. (IP11) The social pressure to be positive seemed evident in one focus group as a participant who complained about ICM SEs was met with admonishment from other group members, one of whom stated, “At least you're alive.” (FG1P4).

### 3.6 Acceptance and adaptation

Some participants recounted how changes in their lifestyle, such as cutting back on work or retiring early, made it easier to manage ICM SEs. Others described accepting their SEs and adapting to the limitations they experienced, “I would reflect on how it was and how I used to do it, and this is how I am doing it now…I knew I could do it better … do it quicker but I can't now and I came to peace and to terms with that.” (IP8) As one patient responded, when asked if the multiple ostomies resulting from the SEs had an impact on daily life, “Oh no, I just carried on. I had to cut back on some of the physical activities for sure, but other than that you just make do. You carry on.” (IP16) Another young participant described how he imagined himself as being retired in order to come to terms with the limited functioning brought about by his ICM SEs.

### 3.7 Feeling supported

Most patients expressed a high degree of confidence in their treatment team, describing their doctors as “brilliant” and “marvelous” (IP4, IP8, IP17). One participant said that if he experienced severe SEs or if his ICM treatment ultimately did not work, he was certain that his doctor would have another treatment option. Nurses and other team members were also described as highly competent, caring, supportive, and attentive; their constant monitoring of patients for the slightest change in their physical symptoms was described as reassuring. Many patients also spoke of receiving practical and emotional support from family, employers, and friends over the course of their treatment, which they characterized as invaluable. As one commented, “I've been lucky to have had very good support. My friends really rallied around. When I had to go to treatment they offered to take me…I have a lot of friends now that will call to find out how I am doing. I also have a supportive family that are there to help if I need it.” (FG3P13).

### 3.8 Faith in medical innovation

A number of patients expressed interest, excitement, and confidence in the medical innovation that ICM treatment represented. They said they felt incredibly lucky to have access to such “cutting edge treatment” (FG3P14) and/or to be deemed eligible to participate in a clinical trial. Immunotherapy was seen by some as the “way of the future” (IP16) in cancer treatment and its mode of action was perceived as more natural and less toxic than traditional treatments such as chemotherapy and radiation. As one patient stated, “It's a miracle the way it works because it's a whole new breakthrough; it's not attacking the cancer, it's energizing my own immune system to do it.” (IP19). ICM treatment sparked a renewed faith and enthusiasm about scientific innovation and many seemed certain that another breakthrough was imminent. Patients expressed pride in contributing to the future; as one described, “The fact that this can help thousands of other people is on your mind. You really feel good about it.” (IP10).

## 4 DISCUSSION

To our knowledge this is the first study of patients' accounts of their lived experiences with ICM treatment and the impact of related SEs on their well‐being. Despite immunotherapy becoming a standard of care for many tumor types, relatively little is known about how it is actually experienced by patients. We identified seven major categories of SEs and by exploring how they were experienced within the lives of patients we demonstrated their impact upon various domains of their HRQOL. SEs had not only physical impact (eg, pain) but also functional, emotional, and social effects on participants as well. We reported on the complex ways in which patients' experiences with ICM‐related SEs intersected with cancer‐related illness, prior and current treatments, their understanding and interpretation of the role and mechanism(s) of action of ICM therapy, and the implications that SEs had on different aspects of their lives. We identified heterogeneity in terms of how participants could experience the same SEs very differently (eg, in terms of intensity and duration); the different constellations of SEs for each individual; a wide diversity of SEs; and the range of impacts these SEs could have, from negligible or nonexistent to severe and life threatening.

As mentioned previously this study is part of a broader series of investigations undertaken to develop a scale to measure the impacts that ICM treatment and its related SEs has on HRQOL.^10^ This scale (the FACT‐ICM) will be incorporated into the FACIT measurement system^27^ and once validated may be implemented in trials and clinical practice to evaluate HRQOL outcomes in patients receiving ICMs. By providing the only in depth account of patients' first‐hand experiences with ICM treatment and its SEs, the present study served as the essential first step in the process of developing this treatment toxicity subscale. Some of the items that were considered for inclusion in this subscale were originally generated from the patient interviews recounted here, but potential items were also based on the results of a systematic review, as well as surveys and interviews conducted with physicians experienced in treating patients with ICMs.^10^ This process of item generation, reduction, and scale refinement is reported elsewhere in a separate publication.^10^ Furthermore this work explained for the first time the link between ICM‐related SEs and HRQOL in patients treated with ICMs.

The main categories of SEs we identified corroborate what has been reported from a variety of clinical trials in terms of type and frequency of SEs related to ICMs such as fatigue and GI toxicities.^5^, ^6^, ^7^ In addition, we have identified important negative impacts of other types of SEs such as cutaneous symptoms and those related to metabolism and nutrition; the impact of these types of SEs can be underappreciated by clinicians and are typically not covered by general HRQOL tools such as the FACT‐G and the EORTC QLQC30.^28^, ^29^ Our findings with regard to living with uncertainty, and its emotional impact, also resonate with previous studies on patients dealing with chronic illness and encountering new medical interventions, and this research could inform strategies for managing patient uncertainty in relation to ICMs such as patient education and knowledge translation initatives.^22^, ^30^, ^31^

While this is the first paper to detail the experiences of patients receiving ICMs, it echoes prior literature more broadly for cancer patients on treatment. For example, the acceptance and adaptation of patients to their SEs by minimizing them and/or reframing their causes, severity and meaning was expected in light of prior research.^22^, ^31^, ^32^ Similarly, faith in medical innovation, gratitude for social support, and focus on survival (vs HRQOL), hope, and positive thinking are also consistent with previous research.^18^, ^33^, ^34^, ^35^ Favorable accounts provided by cancer patients may be driven by an expectation of positivity that has been created by cancer survivorship movements.^33^, ^34^, ^35^ Furthermore, patients who participate in research are more likely to rate their care and treatment as good to excellent and to describe their experiences in a positive manner.^36^

There are several limitations to our study. All the patients were recruited from a single center, which may have influenced their accounts. Patients may have also been influenced by the desire to portray their treatment and medical team in a positive light. Notably, the clinicians who approached patients to participate were not involved in conducting the focus groups or interviews; and patients were informed of the confidential and anonymous nature of their involvement. However, they may still have believed that there was a possibility that their input could be seen and identified by their treatment team. While the study's qualitative methodology and relatively small sample size may limit the potential generalizability of our findings, our objective of this initial part of the study was to collect first‐hand information from patients about their ICM treatment. A qualitative study such as ours is not intended to be generalizable to an entire population but to provide for an in depth analysis of individual experiences.^37^ It afforded us an opportunity to explore patient experiences with ICM treatment in depth and elucidated the myriad of complex ways in which ICMs impacted their lives.

## 5 AUTHOR CONTRIBUTION STATEMENT

The following authors have made substantial contributions: KA‐L, JP, and ARH were involved in the conception OR design of the work OR drafted the manuscript**.** KA‐L, NB, CM, JP, and ARH were involved in the acquisition, analysis, or interpretation of data**.** All authors approved the submitted version. All authors agreed both to be personally accountable for the author's own contributions and to ensure that questions related to the accuracy or integrity of any part of the work, are appropriately investigated, resolved, and the resolution documented in the literature..

## 6 CONFLICT OF INTEREST STATEMENT

The authors do not have any conflict of interests related to this manuscript.

## Data Availability

The data that support the findings of this study are available from the corresponding author upon reasonable request.

## References

[1] Hargadon KM , Johnson CE , Williams CJ . Immune checkpoint blockade therapy for cancer: An overview of FDA‐approved immune checkpoint inhibitors. Int Immunopharmacol. 2018;62:29‐39. 29990692 10.1016/j.intimp.2018.06.001

[2] National Cancer Institute Staff . FDA Approvals ‐ Cancer Currents Blog. Available from URL: http://www.cancer.gov/news-events/cancer-currents-blog/fda-approvals [accessed 28 March 2019].

[3] Harrow A , Dryden R , McCowan C , et al. A hard pill to swallow: a qualitative study of women's experiences of adjuvant endocrine therapy for breast cancer. BMJ Open. 2014;4(6):e005285‐e005285. 10.1136/bmjopen-2014-005285 PMC4067895 24928595

[4] Coolbrandt A , Dierckx de Casterlé B , Wildiers H , et al. Dealing with chemotherapy‐related symptoms at home: a qualitative study in adult patients with cancer. Eur J Cancer Care. 2016;25:79‐92. 10.1111/ecc.12303 25752741

[5] Postow M , Sidlow R , Hellmann M . Immune‐related adverse events associated with immune checkpoint blockade. N Engl J Med. 2018;378:158‐168. 29320654 10.1056/NEJMra1703481

[6] Day D , Hansen A . Immune‐related adverse events associated with immune checkpoint inhibitors. BioDrugs. 2016;30:571‐584. 27848165 10.1007/s40259-016-0204-3

[7] Khoja L , Day D , Wei‐Wu Chen T , Siu L , Hansen A . Tumour and class‐specific patterns of immune‐related adverse events of immune checkpoint inhibitors: a systematic review. Ann Oncol. 2017;28:2377‐2385. 28945858 10.1093/annonc/mdx286

[8] Champiat S , Lambotte O , Barreau E , et al. Management of immune checkpoint blockade dysimmune toxicities: a collaborative position paper. Ann Oncol. 2016;27:559‐574. 26715621 10.1093/annonc/mdv623

[9] Spain L , Diem S , Larkin J . Management of toxicities of immune checkpoint inhibitors. Cancer Treat Rev. 2016;44:51‐60. 26874776 10.1016/j.ctrv.2016.02.001

[10] Hansen AR , Ala‐Leppilampi K , McKillop C , et al. Development of the Functional Assessment of Cancer Therapy‐Immune Checkpoint Modulator (FACT‐ICM): a toxicity subscale to measure quality of life in cancer patients treated with ICMs. Ann Oncol. 2019;30:v475‐v532. 10.1002/cncr.32692 31914209

[11] Denzin NK , Lincoln YS . Handbook of Qualitative Research (2nd Ed.): The discipline and practice of qualitative research. Thousand Oaks: Sage Publications Inc, 2000:1‐29.

[12] Thorne S , Kirkham SR , MacDonald‐Emes J . Interpretive description: a noncategorical qualitative alternative for developing nursing knowledge. Res Nurs Health. 1997;20:169‐177. 9100747 10.1002/(sici)1098-240x(199704)20:2<169::aid-nur9>3.0.co;2-i

[13] Thorne S , Kirkham SR , O’Flynne‐Magee K . The analytic challenge in interpretive description. Int J Qualitative Methods. 2004;3:1‐11.

[14] Kuzel AJ . Sampling in qualitative inquiry In: Crabtree BF, Miller WL, eds. Doing qualitative research Thousand Oaks. CA: Sage; 1992:31‐44.

[15] Green J , Thorogood N . Qualitative methods for health research. London: Sage; 2004.

[16] Patton M . Enhancing the quality and credibilty of qualitative analysis. Health Serv Res. 2000;34:1189‐1208. PMC1089059 10591279

[17] Strauss A , Corbin J . Basics of qualitative research: Techniques and procedures for developing grounded theory. Thousand Oaks, CA: Sage; 1990.

[18] Luoma ML , Hakamies‐Blomqvist L . The meaning of quality of life in patients being treated for advanced breast cancer: a qualitative study. Psycho‐Oncol. 2004;13:729‐739. 10.1002/pon.788 15386642

[19] Hennink M , Hutter I , Bailey A . Qualitative research methods, 1st edn Thousand Oaks, CA: Sage; 2011.

[20] Vogl S . Telephone versus face‐to‐face interviews: mode effect on semistructured interviews with children. Sociol Methodol. 2013;43:133‐177.

[21] Novick G . Is there a bias against telephone interviews in qualitative research? Res Nurs Health. 2008;31:391‐398. 18203128 10.1002/nur.20259 PMC3238794

[22] Davis M , Frankis J , Flowers P . Uncertainty and ‘technological horizon’ in qualitative interviews about HIV treatment. Health. 2006;10:323‐344. 16775018 10.1177/1363459306064489

[23] Thorne S . Interpretive description: Qualitative research for applied practice. New York, NY: Routledge; 2016.

[24] van Manen M . Researching lived experience: Human science for an action sensitive pedagogy. New York, NY: University of New York Press; 1990.

[25] The PB . Zoom model: a dynamic framework for the analysis of life histories. Qual Inq. 1999;5:393‐410.

[26] Butler‐Kisber L . Qualitative inquiry: thematic, narrative and arts informed perspectives. London: Sage; 2010.

[27] Functional assessment of chronic illness therapy. Questionnaires. Available from URL: http://www.facit.org [accessed March 2019].

[28] Cella DF , Tulsky DS , Gray G , et al. The functional assessment of cancer therapy scale: development and validation of the general measure. J Clin Oncol. 1993;11:570‐579. 8445433 10.1200/JCO.1993.11.3.570

[29] Aaronson NK , Ahmedzai S , Bergman B , et al. The European Organization for Research and Treatment of Cancer QLQ‐C30: a quality‐of‐life instrument for use in international clinical trials in oncology. J Natl Cancer Inst. 1993;85:365‐376. 8433390 10.1093/jnci/85.5.365

[30] Hinton D , Kirk S . Living with uncertainty and hope: a qualitative study exploring parents’ experiences of living with childhood multiple sclerosis. Chronic Illness. 2017;13:88‐99. 27539955 10.1177/1742395316664959

[31] Kvåle K , Synnes O . Living with life‐prolonging chemotherapy—control and meaning‐making in the tension between life and death. Eur J Cancer Care. 2018;27(1):e12770. 10.1111/ecc.12770 28892215

[32] Korfage IJ , de Koning HJ , Essink‐Bot ML . Response shift due to diagnosis and primary treatment of localized prostate cancer: a then‐test and a vignette study. Qual Life Res. 2007;16:1627‐1634. 17917793 10.1007/s11136-007-9265-6 PMC2062490

[33] Trusson D , Pilnick A . Between stigma and pink positivity: women's perceptions of social interactions during and after breast cancer treatment. Sociol Health Illn. 2017;39:458‐473. 27577849 10.1111/1467-9566.12486

[34] Pertl MM , Quigley J , Hevey D . 'I'm not complaining because I'm alive': barriers to the emergence of a discourse of cancer‐related fatigue. Psychol Health. 2014;29:141‐161. 24070024 10.1080/08870446.2013.839792

[35] Kaiser K . The meaning of the survivor identity for women with breast cancer. Soc Sci Med. 2008;67:79‐87. 18450347 10.1016/j.socscimed.2008.03.036 PMC2494568

[36] McGrath‐Lone L , Ward H , Schoenborn C , Day S . The effects of cancer research participation on patient experience: a mixed‐methods analysis. Eur J Cancer Care (Engl). 2016;25:1056‐1064. 26094639 10.1111/ecc.12336 PMC5095768

[37] Tracy SJ . Qualitative quality: Eight “Big‐tent” criteria for excellent qualitative research. Qual Inq. 2010;16:837‐851.

